# Impact of COVID-19 on incidence and outcomes of post-infarction mechanical complications in Europe

**DOI:** 10.1093/icvts/ivad198

**Published:** 2023-12-18

**Authors:** Daniele Ronco, Matteo Matteucci, Justine Mafalda Ravaux, Mariusz Kowalewski, Giulio Massimi, Federica Torchio, Cinzia Trumello, Shiho Naito, Nikolaos Bonaros, Michele De Bonis, Dario Fina, Adam Kowalówka, Marek Deja, Federica Jiritano, Giuseppe Filiberto Serraino, Jurij Matija Kalisnik, Carlo De Vincentiis, Marco Ranucci, Theodor Fischlein, Claudio Francesco Russo, Massimiliano Carrozzini, Udo Boeken, Nikolaos Kalampokas, Michele Golino, Roberto De Ponti, Matteo Pozzi, Jean-François Obadia, Matthias Thielmann, Roberto Scrofani, Stefania Blasi, Giovanni Troise, Carlo Antona, Andrea De Martino, Giosuè Falcetta, Guglielmo Actis Dato, Paolo Severgnini, Andrea Musazzi, Roberto Lorusso

**Affiliations:** Department of Cardiothoracic Surgery, Heart and Vascular Centre, Maastricht University Medical Centre, Maastricht, Netherlands; Department of Medicine and Surgery, Circolo Hospital, University of Insubria, Varese, Italy; Thoracic Research Centre, Collegium Medicum Nicolaus Copernicus University, Innovative Medical Forum, Bydgoszcz, Poland; Congenital Cardiac Surgery Department, IRCCS Policlinico San Donato, San Donato Milanese, Italy; Cardiac Surgery Unit, Cardio-Thoraco-Vascular Department, Niguarda Hospital, Milan, Italy; Department of Cardiothoracic Surgery, Heart and Vascular Centre, Maastricht University Medical Centre, Maastricht, Netherlands; Department of Medicine and Surgery, Circolo Hospital, University of Insubria, Varese, Italy; Thoracic Research Centre, Collegium Medicum Nicolaus Copernicus University, Innovative Medical Forum, Bydgoszcz, Poland; Department of Cardiothoracic Surgery, Heart and Vascular Centre, Maastricht University Medical Centre, Maastricht, Netherlands; Department of Cardiothoracic Surgery, Heart and Vascular Centre, Maastricht University Medical Centre, Maastricht, Netherlands; Thoracic Research Centre, Collegium Medicum Nicolaus Copernicus University, Innovative Medical Forum, Bydgoszcz, Poland; Department of Cardiac Surgery, Central Clinical Hospital of the Ministry of Interior, Centre of Postgraduate Medical Education, Warsaw, Poland; Department for the Treatment and Study of Cardiothoracic Diseases and Cardiothoracic Transplantation, IRCCS-ISMETT, Palermo, Italy; Department of Cardiothoracic Surgery, Heart and Vascular Centre, Maastricht University Medical Centre, Maastricht, Netherlands; Thoracic Research Centre, Collegium Medicum Nicolaus Copernicus University, Innovative Medical Forum, Bydgoszcz, Poland; Cardiac Surgery Unit, Cardio-Thoraco-Vascular Department, Niguarda Hospital, Milan, Italy; Department of Medicine and Surgery, Circolo Hospital, University of Insubria, Varese, Italy; Cardiac Surgery Unit, IRCCS Policlinico San Donato, San Donato Milanese, Italy; Cardiothoracic Surgery Department, San Raffaele University Hospital, Milan, Italy; Department of Cardiovascular Surgery, University Heart & Vascular Center Hamburg, Hamburg, Germany; Department of Cardiac Surgery, Medical University of Innsbruck, Innsbruck, Austria; Cardiothoracic Surgery Department, San Raffaele University Hospital, Milan, Italy; Department of Cardiothoracic Surgery, Heart and Vascular Centre, Maastricht University Medical Centre, Maastricht, Netherlands; Department of Cardiology, Città di Lecce Hospital, GVM Care and Research, Lecce, Italy; Thoracic Research Centre, Collegium Medicum Nicolaus Copernicus University, Innovative Medical Forum, Bydgoszcz, Poland; Department of Cardiac Surgery, Medical University of Silesia, School of Medicine in Katowice, Katowice, Poland; Department of Cardiac Surgery, Upper-Silesian Heart Center, Katowice, Poland; Department of Cardiac Surgery, Medical University of Silesia, School of Medicine in Katowice, Katowice, Poland; Department of Cardiac Surgery, Upper-Silesian Heart Center, Katowice, Poland; Department of Cardiothoracic Surgery, Heart and Vascular Centre, Maastricht University Medical Centre, Maastricht, Netherlands; Department of Experimental and Clinical Medicine, “Magna Graecia” University of Catanzaro, Catanzaro, Italy; Department of Experimental and Clinical Medicine, “Magna Graecia” University of Catanzaro, Catanzaro, Italy; Department of Cardiac Surgery, Cardiovascular Center, Klinikum Nürnberg, Paracelsus Medical University, Nuremberg, Germany; Cardiac Surgery Unit, IRCCS Policlinico San Donato, San Donato Milanese, Italy; Department of Cardiovascular Anesthesia and Intensive Care, IRCCS Policlinico San Donato, San Donato Milanese, Italy; Department of Cardiac Surgery, Cardiovascular Center, Klinikum Nürnberg, Paracelsus Medical University, Nuremberg, Germany; Cardiac Surgery Unit, Cardio-Thoraco-Vascular Department, Niguarda Hospital, Milan, Italy; Cardiac Surgery Unit, Cardio-Thoraco-Vascular Department, Niguarda Hospital, Milan, Italy; Department of Cardiovascular Surgery, University Hospital Düsseldorf, Heinrich Heine University, Düsseldorf, Germany; Department of Cardiovascular Surgery, University Hospital Düsseldorf, Heinrich Heine University, Düsseldorf, Germany; Department of Medicine and Surgery, Circolo Hospital, University of Insubria, Varese, Italy; Department of Medicine and Surgery, Circolo Hospital, University of Insubria, Varese, Italy; Department of Cardiac Surgery, Louis Pradel Cardiologic Hospital, Lyon, France; Department of Cardiac Surgery, Louis Pradel Cardiologic Hospital, Lyon, France; Department of Thoracic and Cardiovascular Surgery, West-German Heart Center, University of Duisburg-Essen, Essen, Germany; Cardiac Surgery Unit, Fondazione IRCCS Ca’ Granda, Ospedale Maggiore Policlinico, Milan, Italy; Cardiac Surgery Unit, Poliambulanza Foundation Hospital, Brescia, Italy; Cardiac Surgery Unit, Poliambulanza Foundation Hospital, Brescia, Italy; Cardiac Surgery Unit, Fondazione IRCCS Ca’ Granda, Ospedale Maggiore Policlinico, Milan, Italy; Section of Cardiac Surgery, University Hospital, Pisa, Italy; Section of Cardiac Surgery, University Hospital, Pisa, Italy; Cardiac Surgery Department, Mauriziano Hospital, Turin, Italy; Department of Biotechnology and Sciences of Life, Circolo Hospital, University of Insubria, Varese, Italy; Cardiac Surgery Unit, Circolo Hospital, Varese, Italy; Department of Cardiothoracic Surgery, Heart and Vascular Centre, Maastricht University Medical Centre, Maastricht, Netherlands; Cardiovascular Research Institute Maastricht (CARIM), Maastricht, Netherlands

**Keywords:** Acute myocardial infarction, Cardiac rupture, Ventricular septal rupture, Papillary muscle rupture, COVID-19

## Abstract

**OBJECTIVES:**

Post-acute myocardial infarction mechanical complications (post-AMI MCs) represent rare but life-threatening conditions, including free-wall rupture, ventricular septal rupture and papillary muscle rupture. During the coronavirus disease-19 (COVID-19) pandemic, an overwhelming pressure on healthcare systems led to delayed and potentially suboptimal treatments for time-dependent conditions. As AMI-related hospitalizations decreased, limited information is available whether higher rates of post-AMI MCs and related deaths occurred in this setting. This study was aimed to assess how COVID-19 in Europe has impacted the incidence, treatment and outcome of MCs.

**METHODS:**

The CAUTION-COVID19 study is a multicentre retrospective study collecting 175 patients with post-AMI MCs in 18 centres from 6 European countries, aimed to compare the incidence of such events, related patients’ characteristics, and outcomes, between the first year of pandemic and the 2 previous years.

**RESULTS:**

A non-significant increase in MCs was observed [odds ratio (OR) = 1.15, 95% confidence interval (CI) 0.85–1.57; *P *=* *0.364], with stronger growth in ventricular septal rupture diagnoses (OR = 1.43, 95% CI 0.95–2.18; *P *=* *0.090). No significant differences in treatment types and mortality were found between the 2 periods. In-hospital mortality was 50.9% and was higher for conservatively managed cases (90.9%) and lower for surgical patients (44.0%). Patients admitted during COVID-19 more frequently had late-presenting infarction (OR = 2.47, 95% CI 1.24–4.92; *P *=* *0.010), more stable conditions (OR = 2.61, 95% CI 1.27–5.35; *P *=* *0.009) and higher EuroSCORE II (OR = 1.04, 95% CI 1.01–1.06; *P *=* *0.006).

**CONCLUSIONS:**

A non-significant increase in MCs incidence occurred during the first year of COVID-19, characterized by a significantly higher rate of late-presenting infarction, stable conditions and EuroSCORE-II if compared to pre-pandemic data, without affecting treatment and mortality.

**Figure:**
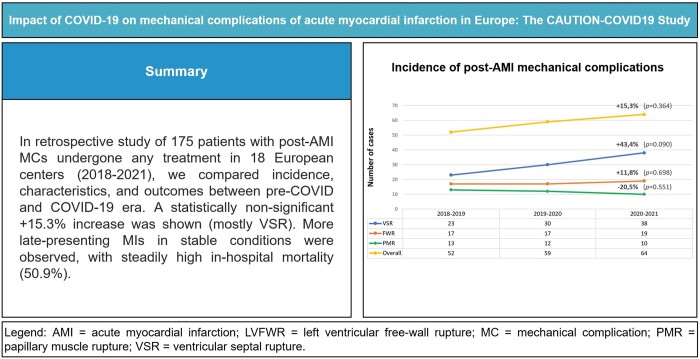

## INTRODUCTION

Post-acute myocardial infarction mechanical complications (post-AMI MCs) represent rare, but potentially catastrophic events, including ventricular septal rupture (VSR), left ventricular free-wall rupture (LVFWR) and papillary muscle rupture (PMR) [1]. During the past decades, the incidence of post-AMI MCs had significantly decreased, mostly due to the improvements and wide diffusion of early percutaneous revascularization strategies, namely thrombolysis and percutaneous coronary intervention [1, 2]. However, the coronavirus disease-19 (COVID-19) pandemic has brought about some relevant, unexpected changes in the healthcare systems capability throughout Europe to properly manage time-dependent diseases, including AMI and related complications [3]. Indeed, as hospitals have been overwhelmed by COVID-19 patients, subjects affected by other acute conditions could not receive the appropriate treatment in due time. Therefore, a lower rate of AMI hospitalizations, paralleled by higher rates of complications and mortality related to such condition, have been reported [3–7]. Similarly, an increasing trend of post-AMI MCs occurrence has been observed by various investigators [7–9].

We conducted an international, multicentre, retrospective study on post-AMI MCs during the COVID-19 pandemic (CAUTION-COVID19 study) and a limited time just prior to it. The investigation herein reported was specifically designed to assess whether COVID-19 outbreak in Europe has somehow impacted the incidence, treatment, and outcome of MCs, as well as patients’ characteristics.

## PATIENTS AND METHODS

### Ethics statement

The study protocol was authorized by the local ethical committees of each centre (core centre approval: IT-VA:85/2021). Informed consent was waived because of the retrospective nature of the study.

### Patient population and study design

**Figure 1:**
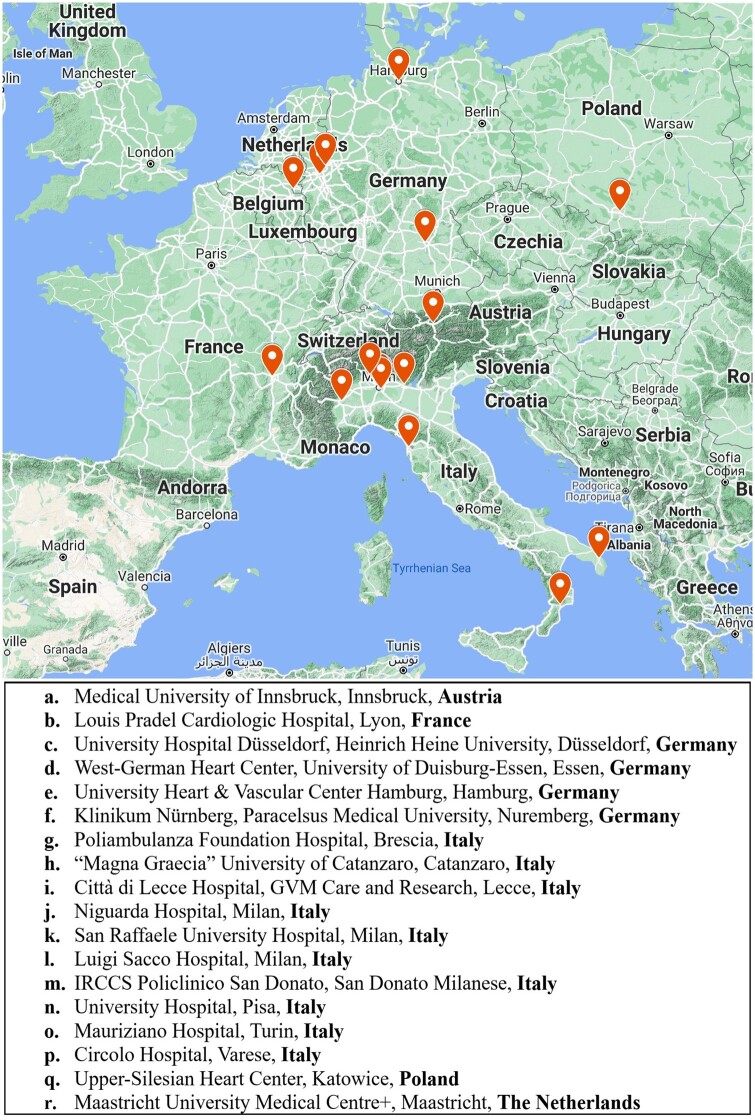
Distribution of European centres enrolled in the study.

The patients were recruited from the database of the CAUTION-COVID19 study (‘Mechanical complications of acute myocardial infarction: an international multicentre cohort study during COVID-19 pandemic among hospitalized patients’). The CAUTION-COVID19 study (trial registration: Clinicaltrials.gov, NCT04813692) is a retrospective, multicentre, observational trial aimed at evaluating the incidence, treatment and outcomes of post-AMI MCs during the first year of the COVID-19 pandemic with respect to the 2 previous years. The herein reported study included all the adult patients (aged >18 years) who were hospitalized with a diagnosis of post-AMI MCs, independently of the treatment received (i.e. surgical, percutaneous or conservative), between 1 March 2018 and 28 February 2021 in 18 different centres from 6 European countries (Austria, France, Germany, Italy, Poland and the Netherlands), as presented in Fig. 1. Patients have been identified through diagnosis-specific ICD-9 codes, administrative databases or surgical records.

The study was conducted in accordance with the guidelines of the Declaration of Helsinki for patient data use and evaluation. A unified patient dataset was used to collect pertinent information, including demographic characteristics, clinical history, diagnostic workup, type of treatment and operative and postoperative data, as well as information on outcomes, from medical records. This report follows the STROBE reporting guidelines for observational studies.

### Definitions and endpoints

History of myocardial infarction (MI) refers to a documented previous episode of AMI unrelated to the current ischaemic event, independently from the time of occurrence. Late-presenting MI was diagnosed when >48 h passed from the onset of AMI-related symptoms to hospital presentation. Hybrid/staged procedure referred to patients undergone both surgical and percutaneous treatments, independent of timing and cause. Urgent operation was an operation required during the same hospitalization for patients not electively admitted. Emergent operation was an operation occurred within 24 h, while salvage operation was identified when patients required cardiopulmonary resuscitation while going to the operating room. Patients managed conservatively were also included.

Rupture types and repair techniques for each subtype of post-AMI MC have been previously defined [10–12]. In-hospital (early) mortality was defined as all-cause death occurring within 30 days from the intervention, or during the same hospitalization, regardless of the treatment. Intraoperative mortality was defined as death occurred during the operation.

The primary endpoint was the incidence of post-infarction MCs during the first year of COVID-19 pandemic compared with the previous 2 years. The secondary endpoint was early mortality, according to different treatments.

### Statistical analysis

Continuous variables have been tested for normality distribution with the Shapiro–Wilk test and are reported as mean ± standard deviation (variables not violating the normality assumption) or median with interquartile range (variables violating the normality assumption). Categorical variables were reported as frequencies with percentages. Multiple imputation was performed for all missing values, including only variables presenting ≤30% of missing data. Continuous variables were compared individually with the Student’s *t*-test or the Mann–Whitney *U*-test, while categorical variables were tested with the χ^2^ or Fisher’s exact test, as appropriate. Subsequently, variables of clinical interest that achieved a *P*-value <0.10 at univariate analysis, were tested for multicollinearity and then entered into a multivariate logistic regression analysis, to identify independent variables characterizing the patients hospitalized during COVID-19, compared to the pre-COVID-19 era. Incidence rate ratios comparing the years of the study period were calculated through the Poisson regression. Data analyses were performed using SPSS Statistics 26.0 (IBM Corp., Armonk, NY, USA). A 2-tailed *P*-value <0.05 was considered statistically significant.

## RESULTS

A total of 175 patients were included in this study, specifically 87 (49.7%) with VSR, 49 (28.0%) with LVFWR, 35 (20.0%) with PMR and 4 (2.3%) with concomitant VSR and LVFWR. Patients’ baseline characteristics on hospital admission are presented in Supplementary Material, Table S1. Median age was 70.0 years, with male predominance. Apart from hypertension, all the other comorbidities were found in less than half of subjects. Importantly, <20% of patients had a history of MI and less than one-fourth had a previous revascularization.

Most patients (87.4%) were admitted with ST-elevation MI. Only one-third of subjects were haemodynamically stable, whereas almost 50% were in cardiogenic shock and nearly one-sixth were in cardiac arrest. Notwithstanding, almost 60% of the subjects presented during AMI. Preoperative coronarography was performed in almost 10% of patients, showing mostly multivessel disease, and >50% of subjects underwent a percutaneous revascularization. A mechanical circulatory support device has been implanted in about half of patients, including intra-aortic balloon pump (IABP) in 69 (39.4%) subjects, extracorporeal membrane oxygenation (ECMO) in 27 (15.4%) and Impella in 9 (5.1%) of them.

The median time from the onset of AMI symptoms to hospital admission was 72.0 h and the diagnosis was achieved almost always early after admission; for operated patients, the median time from MC diagnosis to operation was 60.0 h.

### Treatment of post-AMI MCs

Most patients underwent surgery (80.6%), while 6 patients received percutaneous closure and 6 had a hybrid/staged procedure, almost all belonging to the VSR group (11, 91.7%). Emergent or salvage intervention was performed in almost half of the cases. Only 22 patients (12.6%), mostly belonging to LVFWR group, were treated conservatively, mostly for advanced heart failure and severely compromised conditions that contraindicated any treatment other than palliative care.

Operative and perioperative variables for operated patients are presented in Supplementary Material, Table S2. Concomitant coronary artery bypass grafting during surgical repair was performed in <50% of subjects. Interestingly, one patient had already undergone off-pump coronary artery bypass grafting 3 days before VSR occurrence. In 2 patients with LVFWR only sternotomy could be executed under cardiopulmonary resuscitation, but death was declared before any other procedure could be performed.

Postoperatively, almost 60% of patients were supported with IABP and almost 30% with ECMO. One VSR patient had intraoperative ECMO implantation and was supported until heart transplantation. Median intensive care unit stay was 6.0 days, while the median hospital stay was around 2 weeks.

The commonest complications occurring during hospitalization included low cardiac output syndrome in 34.0% of patients and acute renal failure in 23.1% of cases. Recurrent LVFWR or VSR occurred in 12.9% of subjects.

### In-hospital outcomes

In-hospital outcomes in different subgroups are presented in Supplementary Material, Table S3. Overall, in-hospital mortality was 50.9% (89 patients), with 6 (3.9%) intraoperative deaths, due to incontrollable bleeding (4 cases) and severe biventricular failure making cardiopulmonary bypass weaning impossible (2 cases). The cause of death was most often low cardiac output syndrome followed by multiorgan failure. An LVFWR recurrence was the cause of death in 7 subjects, while fatal recurrent VSR occurred in 3 patients.

A significantly lower mortality was registered for PMR patients (*P *=* *0.003) with respect to VSR and LVFWR. Conversely, almost all patients treated conservatively died during hospitalization (90.9%). In-hospital mortality was not different between the pre-COVID-19 and the COVID-19 era, neither according to the MC type and treatment type, nor considering the whole cohort of patients.

### Pre-COVID-19 versus COVID-19 era

**Figure 2:**
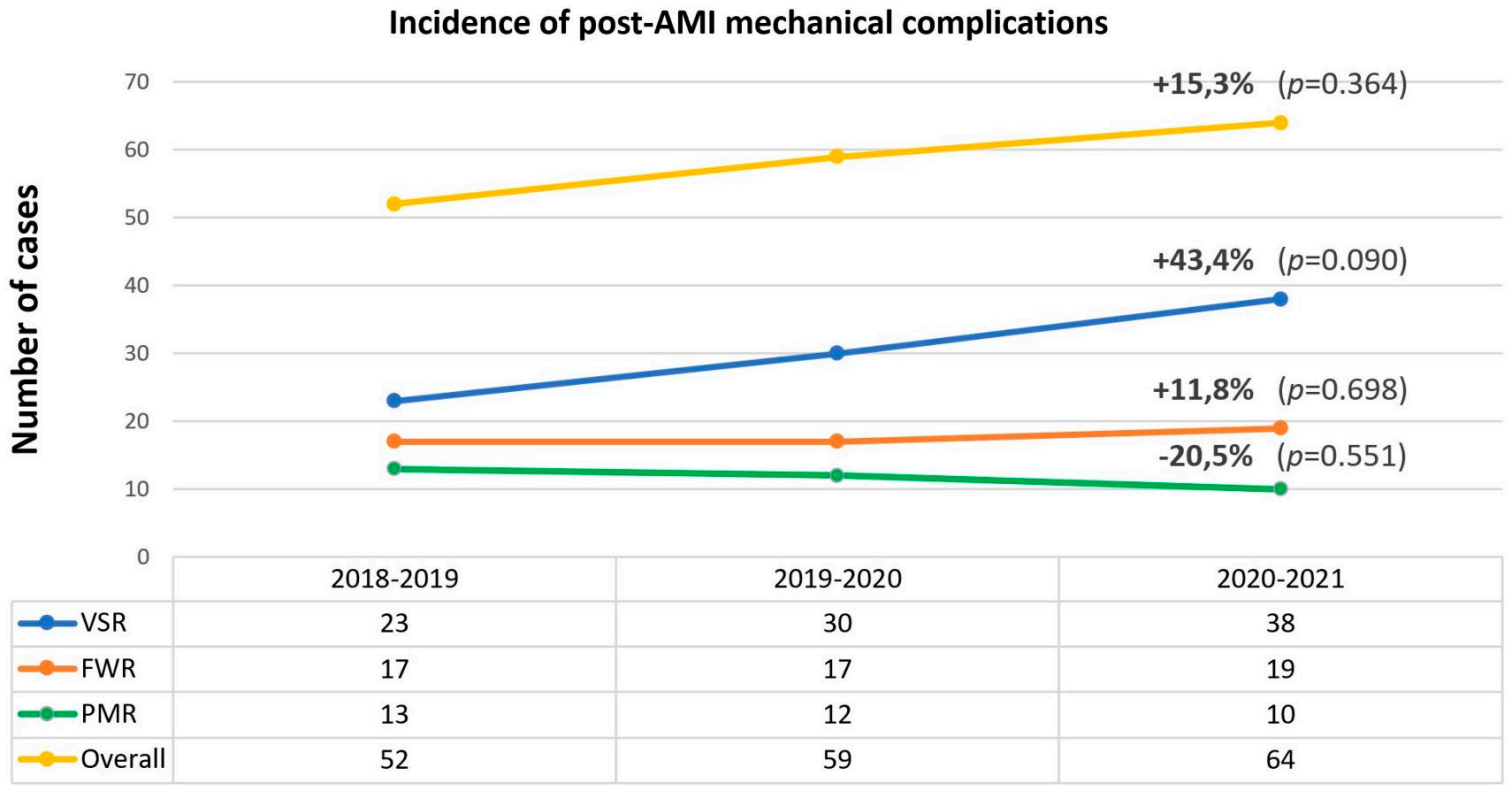
Rate of variation in mechanical complications diagnosis during the first year of COVID-19 pandemic as compared to the 2 previous years. COVID-19: coronavirus disease-19; FWR: free-wall rupture; PMR: papillary muscle rupture; VSR: ventricular septal rupture.

**Figure 3:**
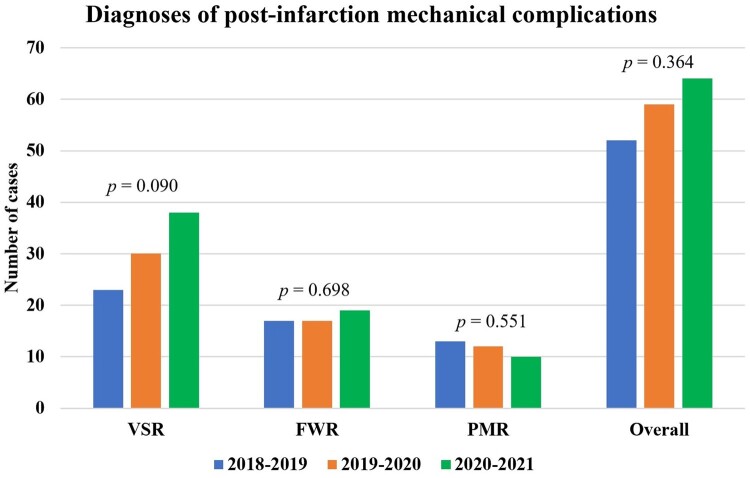
Number of mechanical complications cases during the study period. FWR: free-wall rupture; PMR: papillary muscle rupture; VSR: ventricular septal rupture.

As compared to the previous 2 years, during the first year of COVID-19 pandemic a non-significant, 15.3% increase in post-AMI MCs diagnoses has been observed among the participating centres [odds ratio (OR) 1.15, 95% confidence interval (CI) 0.85–1.57; *P *=* *0.364]. The most marked increase occurred for VSR (OR 1.43, 95% CI 0.95–2.18; *P *=* *0.090), followed by LVFWR (OR 1.12, 95% CI 0.64–1.96; *P *=* *0.698), while a 20.0% decrease in PMR diagnosis was found (OR 0.80, 95% CI 0.38–1.67; *P *=* *0.551), as presented in Figs 2 and 3. Number of yearly diagnoses for each centre are presented in Supplementary Material, Table S4.

During the COVID-19 period only 2 patients were COVID-19-positive on admission, with a mild form of the disease. One patient had PMR and died early after hospitalization due to cardiac arrest, while the other patient had VSR and underwent successful surgical repair. Finally, only one surgically treated LVFWR patient acquired COVID-19 during hospitalization and died for the complications that occurred thereafter.

At univariate analysis, patients admitted during the COVID-19 era had a higher EuroSCORE II (*P *=* *0.042) and were more commonly late-presenting MI than pending AMI (*P *=* *0.003), with respect to the previous years. Nevertheless, they had a slightly higher body mass index (*P *=* *0.096), were more often haemodynamically stable (*P *=* *0.068) and had a lower rate of IABP adoption (*P *=* *0.093). No other relevant differences have been identified. After intervention, patients operated during COVID-19 era showed a slightly longer intensive care unit and hospital stay. Moreover, no significant between-group differences were found analysing the single MC types.

**Figure 4:**
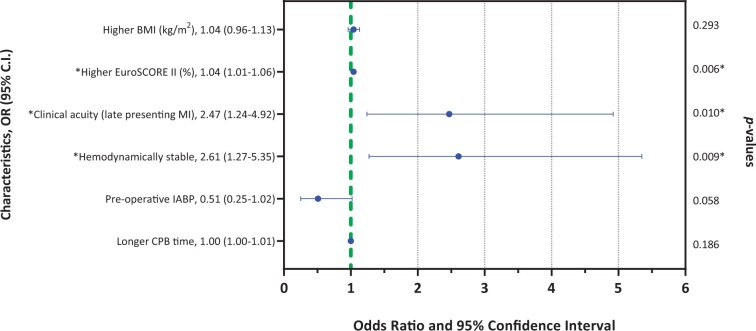
Forest plot of multivariate analysis of difference between pre-COVID-19 and COVID-19 era. Statistically significant for *P*-value <0.05. BMI: body mass index; CI: confidence interval; COVID-19: coronavirus disease-19; CPB: cardiopulmonary bypass; IABP: intra-aortic balloon pump; MI: myocardial infarction; OR: odds ratio.

At multivariate analysis (Fig. 4), patients admitted during the first year of COVID-19 pandemic had a significantly higher EuroSCORE II (OR 1.04, 95% CI 1.01–1.06; *P *=* *0.006), higher rate of late-presenting MI (OR 2.47, 95% CI 1.24–4.92; *P *=* *0.010) and were more often haemodynamically stable (OR 2.61, 95% CI 1.27–5.35; *P *=* *0.009).

## DISCUSSION

Post-AMI MCs represent rare, but potentially lethal complications [1, 13, 14]. Due to the improvements of early percutaneous reperfusion strategies, their incidence has decreased to <1%, remaining stable over the last 2 decades [1, 2, 14]. Since conservative treatment is almost inevitably fatal, especially for LVFWR and VSR, prompt intervention represents the only option [2, 10, 11]. Nevertheless, even timely and adequate treatment had shown unsatisfactory results [11, 14]. Indeed, the recent CAUTION-related investigations have shown that in-hospital mortality has not decreased, identifying post-AMI MCs amongst the most lethal cardiac surgical conditions [10–12].

COVID-19 pandemic has brought an unexpected pressure on healthcare systems worldwide [1, 3, 5]. Accordingly, the hospital capacity in most countries has been overwhelmed by the abruptly increasing COVID-19-related admissions, and some structures have been completely converted into COVID-19 hospitals [15]. Such critical situation has remarkably impacted on the treatment of all the other time-dependent diseases, especially in the first year of pandemic, where systems were under pressure before the possibility to accomplish an effective reorganization [3, 5]. Therefore, for reasons including the patients’ fear of getting COVID-19 infection during hospitalization and the unavailability of hospital beds for non-COVID-19 conditions, patients referral and admission were largely reduced [3, 5]. This occurred in various countries, especially Europe and North America, including a significant decrease in the hospitalizations for AMI and acute heart failure, and resulting in significantly higher mortality rates for these conditions [3–5, 16–22].

As timing of revascularization represents a well-known risk factor for post-AMI MCs occurrence, it is possible that the COVID-19-related situation has impacted also on the number of MCs cases, especially in countries where the standards of care for coronary artery disease are high and COVID-19 has hit hard [7–9]. Such an hypothesis represented the basis of the CAUTION-COVID19 study, meant to compare the first year of COVID-19 pandemic (from the World Health Organization’s declaration of pandemic in March 2020) with the 2 previous years in 18 European centres.

A non-significant increase in the number of post-AMI MCs admissions has been observed, but VSR showed the most relevant growth (approaching 50%). However, it should be considered that our study included only hospital diagnoses, since it was not possible to track all the potentially missed cases of cardiac rupture that could not be hospitalized. Indeed, also a higher rate of out-of-hospital cardiac arrests and deaths has been observed during this period, and it is therefore reasonable to assume that some missed MCs cases could be found among those patients [23, 24]. Moreover, although the remarkable sample size given the rarity of post-AMI MCs and the short timeframe of the current study, the number of patients remains relatively small and may *per se* justify the lack of significance in our results, especially considering the relevant difference observed for VSR patients. Furthermore, it is noteworthy that LVFWR patients represented an unexpectedly high 28% of admissions.

We observed some variations in the trends of diagnosis according to country, region and even hospital within the same region. This may reflect different epidemiological contexts, the relatively asynchronous occurrence of epidemic waves and even the various political decisions taken by different countries to face the epidemic [3, 22]. For instance, within Lombardy region (Northern Italy), cardiac surgical units have been reorganized in hub-and-spoke centres and patients’ referral has significantly changed from centre to centre compared to the previous years [15, 22].

No substantial differences in the baseline characteristics have been found between the 2 periods, nor in comparison to previous reports [10–12]. However, during the first year of pandemic, patients were more often admitted with late-presenting MI and in haemodynamically stable conditions [5, 21]. Although such clinical stability might contrast with most COVID-19 hospital admission policies, this might also explain the lower rate of preoperative IABP implantation, despite a significantly higher EuroSCORE II [25, 26]. Moreover, it is reasonable to hypothesize that during COVID-19 period, patients with rather compensated post-AMI MCs could have access to hospital, despite a suboptimal network of care, while patients with more haemodynamic compromise might have experienced out-of-hospital premature death, thereby enriching the group of uncounted post-AMI MCs cases [23]. Moreover, the median time from symptoms onset to hospital presentation was about 3 days in both periods and was only slightly shorter for LVFWR than for VSR and PMR.

Almost 90% of patients underwent coronarography and the large majority could receive either percutaneous or surgical revascularization [10–12]. More than half of the subjects presented in cardiogenic shock, as expected. Interestingly, while IABP implantation rate was relatively low, in the 3 years examined for the study, there was a promisingly increased adoption of preoperative and perioperative ECMO, with respect to the CAUTION reports considering the previous 2 decades [10–12]. The increased adoption rate was confirmed in the COVID-19 era, despite the potential shortage of ECMO devices due to their large use in most critical COVID-19 patients [27]. Indeed, although remaining <30% of cases, such a growing adoption might suggest a general shift towards higher rates of mechanical circulatory support applications aimed at reaching patients’ stabilization before intervention [28]. Moreover, 5% of patients (mostly VSR) had Impella support, again highlighting a more pronounced attitude towards circulatory assistance in these settings [28].

No difference in the type of treatment was observed between the 2 periods. Conservative treatment was adopted in a few patients (mostly LVFWR), with a mortality exceeding 90%, confirming previous data [13]. On the other hand, percutaneous treatment was adopted most often in VSR patients, showing that alternative therapeutic strategies are progressively finding their space for selected patients, although results were slightly suboptimal, if compared to surgery [29].

Concerning surgically treated patients, global mortality confirmed as high as 44.0% but was slightly lower in the COVID-19 period, probably suggesting also that EuroSCORE II has some limits in predicting mortality in this setting [13, 14, 25, 26]. Moreover, cardiopulmonary bypass time was lower before COVID-19, albeit not justified by additional procedures and not impacting on patients’ outcome, possibly due to unusual operative conditions. Among different MC types, LVFWR was the most lethal, while PMR mortality was significantly lower, confirming the CAUTION results [10–12].

After intervention, >10% of patients developed rupture recurrence, almost double in the VSR group, suggesting the need of additional optimizations in the perioperative management to improve such suboptimal results [28].

Finally, concerning the subjects belonging to the COVID-19 era group (for whom COVID-19 screening test was available), 2 patients tested positive for COVID-19 on hospital admission: one survived and the other didn’t. Even if recent studies have described a significantly higher mortality for COVID-19-positive patients undergoing cardiac surgery, such small numbers make any further conclusion unfeasible [30]. Moreover, only one patient got COVID-19 infection during hospital stay, that later represented the cause of death. Therefore, despite the sometimes-advocated fear of getting COVID-19 on hospitalization during the pandemic peak, it is reasonable to conclude that, with proper safety measures, quickly reaching a tertiary centre in the presence of complicated AMI, does not represent an additional risk for hospital-acquired infection [9].

### Limitations

Several limitations should be acknowledged for the current study. First, due to the retrospective design, we could not control and examine all potential confounders. The multicentre design of the study required a limited number of collected variables, to limit the amount of missing data, that were anyway handled with multiple imputation. Therefore, the possibility that non-registered variables could have influenced the results cannot be completely ruled out. However, considering the short timeframe and the accurate selection of relevant data, such confounders have been as limited as possible. Nonetheless, the number of centres enrolled per country was widely variable, and the number of patients from each centre is still relatively small due to the low incidence of the diseases, thus potentially influencing the significance of our results. Moreover, we acknowledge that such situation may have varied in the following months due to changing healthcare system organization. Finally, only considering hospital diagnosis of post-AMI MC, the real incidence of such conditions could have been underestimated due to missing diagnoses of patients died out of hospital.

## CONCLUSIONS

Post-AMI MCs remain rare, but life-threatening events. Despite the overwhelming pressure put by COVID-19 pandemic on the healthcare systems throughout Europe, leading to delayed and suboptimal care for other time-dependent conditions, the significant decrease in hospitalizations due to AMI and acute heart failure has been paralleled by more diagnoses of post-AMI MCs, although varying in different centres; VSR showed the most relevant increase (50%), albeit not statistically significant. However, such data may be underestimated by the higher rates of out-of-hospital cardiac-related deaths reported during COVID-19 pandemic. Patients admitted during the COVID-19 period were more frequently late-presenting MI, in stable conditions, although characterized by a higher EuroSCORE II. However, both the types of treatments (largely surgical) and in-hospital mortality didn’t change significantly between the 2 periods. Post-AMI MCs confirm to be almost inevitably lethal when treated conservatively. Finally, the incidence of COVID-19 infection during hospitalization appears to represent a remote occurrence that should not keep patients away from seeking medical care, when facing acute cardiovascular events for prompt diagnosis and appropriate treatment.

## Supplementary Material

ivad198_Supplementary_Data

## Data Availability

The data underlying this article will be shared on reasonable request to the corresponding author.

## ABBREVIATIONS

AMI: Acute myocardial infarction
CI: Confidence interval
COVID-19: Coronavirus disease-19
ECMO: Extracorporeal membrane oxygenation
IABP: Intra-aortic balloon pump
LVFWR: Left ventricular free-wall rupture
MC: Mechanical complication
MI: Myocardial infarction
OR: Odds ratio
PMR: Papillary muscle rupture
VSR: Ventricular septal rupture
