# Supplementary material for: Impact of COVID-19 on incidence and outcomes of post-infarction mechanical complications in Europe
Source: Interdiscip Cardiovasc Thorac Surg. 2023 Dec 18;37(6):ivad198. doi: 10.1093/icvts/ivad198 (PMC10749759; doi:10.1093/icvts/ivad198)
Supplement: ivad198_Supplementary_Data [file ivad198_supplementary_data.docx]

**Supplementary Data**

**Impact of COVID-19 on incidence and outcomes of post-infarction mechanical complications in Europe**

Daniele Ronco, Matteo Matteucci, Justine M. Ravaux, Mariusz Kowalewski, Giulio Massimi, Federica Torchio, Cinzia Trumello, Shiho Naito, Nikolaos Bonaros, Michele De Bonis, Dario Fina, Adam R. Kowalówka, Marek A. Deja, Federica Jiritano, Giuseppe Filiberto Serraino, Jurij Matija Kalisnik, Carlo De Vincentiis, Marco Ranucci, Theodor Fischlein, Claudio Francesco Russo, Massimiliano Carrozzini, Udo Boeken, Nikolaos Kalampokas, Michele Golino, Roberto De Ponti, Matteo Pozzi, Jean-François Obadia, Matthias Thielmann, Roberto Scrofani, Stefania Blasi, Giovanni Troise, Carlo Antona, Andrea De Martino, Giosuè Falcetta, Guglielmo Actis Dato, Paolo Severgnini, Andrea Musazzi, and Roberto Lorusso.

Supplementary Table 1...................................................................................................................pag 2

Supplementary Table 2...................................................................................................................pag 5

Supplementary Table 3...................................................................................................................pag 6

Supplementary Table 4...................................................................................................................pag 7

**Suppl. Table 1. Baseline characteristics on hospital admission (after imputation)**

| **Variable** | **Patients**  (n=175) | **Pre-COVID-19**  (n=111) | **COVID-19 era**  (n=64) | ***p*-value** |
| --- | --- | --- | --- | --- |
| Age *(years)* | 70.0 [61.0-77.0] | 69.0 [61.0-75.0] | 70.0 [61.3-77.0] | 0.572 |
| Sex *(male)* | 109(62.3%) | 68(61.3%) | 41 (64.1%) | 0.713 |
| Body Mass Index *(kg/m^2^)* | 26.1 [23.9-28.7] | 25.8 [23.4-28.4] | 26.7 [24.3-29.3] | ***0.096*** |
| Hypertension | 116 (66.3%) | 77 (69.4%) | 39 (60.9%) | 0.256 |
| Dyslipidemia | 70 (40.0%) | 43 (38.7%) | 27 (42.2%) | 0.654 |
| Diabetes mellitus | 41 (23.4%) | 30 (27.0%) | 11 (17.2%) | 0.139 |
| Smoking habit | 61 (34.9%) | 41 (36.9%) | 20 (31.3%) | 0.447 |
| Chronic obstructive pulmonary disease | 16 (9.1%) | 13 (11.7%) | 3 (4.7%) | 0.120 |
| Chronic kidney disease | 24 (13.7%) | 16 (14.4%) | 8 (12.5%) | 0.723 |
| Cerebrovascular accident | 10 (5.7%) | 7 (6.3%) | 3 (4.7%) | 0.748 |
| Peripheral vascular disease | 14 (8.0%) | 9 (8.1%) | 5 (7.8%) | 0.945 |
| Atrial fibrillation | 26 (14.9%) | 14 (12.6%) | 12 (18.8%) | 0.272 |
| History of MI | 32 (18.3%) | 20 (18.0%) | 12 (18.8%) | 0.904 |
| *Therapy on admission*  Antiplatelet  Anticoagulant  Anti-hypertensive  Antidiabetic  Lipid lowering  Immunosuppressive | 56 (32.0%)  22 (12.6%)  84 (48.0%)  31 (17.7%)  57 (32.6%)  6 (3.4%) | 35 (31.5%)  10 (9.0%)  56 (50.5%)  21 (18.9%)  33 (29.7%)  5 (4.5%) | 21 (32.8%)  12 (18.8%)  28 (43.8%)  10 (15.6%)  24 (37.5%)  1 (1.6%) | 0.819  0.596  0.484  0.676  0.610  0.561 |
| *ECG pattern*  STEMI  NSTEMI | 153 (87.4%)  22 (12.6%) | 94 (84.7%)  17 (15.3%) | 59 (92.2%)  5 (7.8%) | 0.435 |
| NYHA IV | 88 (50.3%) | 53 (47.7%) | 35 (54.7%) | 0.377 |
| EuroSCORE II *(%)* | 18.2 [10.1-27.9] | 17.2 [8.9-24.9] | 20.3 [12.4-32.2] | ***0.042*** |
| *Clinical acuity*  AMI  Late-presenting MI | 104 (59.4%)  71 (40.6%) | 75 (67.6%)  36 (32.4%) | 29 (45.3%)  35 (54.7%) | ***0.003*** |
| *Hemodynamic presentation*  Stable  Chest pain  Pulmonary edema  Cardiogenic shock  Cardiac tamponade  Cardiac arrest | 64 (36.6%)  103 (58.9%)  33 (18.9%)  97 (55.4%)  37 (21.1%)  26 (14.9%) | 35 (31.5%)  61 (55.0%)  18 (16.2%)  61 (55.0%)  23 (20.7%)  16 (14.4%) | 29 (45.3%)  42 (65.5%)  15 (23.4%)  36 (56.3%)  14 (21.9%)  10 (15.6%) | ***0.068***  0.167  0.240  0.868  0.857  0.828 |
| *Mechanical circulatory support*  Intra-aortic balloon pump  ECMO  Impella | 69 (39.4%)  27 (15.4%)  9 (5.1%) | 49 (44.1%)  16 (14.4%)  6 (5.4%) | 20 (31.3%)  11 (17.2%)  3 (4.7%) | ***0.093***  0.625  >0.999 |
| Inotropes | 104 (59.4%) | 63 (56.8%) | 41 (64.1%) | 0.343 |
| Left ventricular ejection fraction *(%)* | 41.0 [35.0-50.0] | 40.0 [35.0-50.0] | 45.0 [35.0-50.0] | 0.865 |
| Coronarography | 153 (87.4%) | 98 (88.3%) | 55 (85.9%) | 0.651 |
| *CAD pattern^a^*  Single-vessel  Multivessel | 61 (39.9%)  92 (60.1%) | 39 (39.8%)  59 (60.2%) | 22 (40.0%)  33 (60.0%) | 0.307 |
| Percutaneous revascularization | 89 (50.9%) | 54 (48.6%) | 35 (54.7%) | 0.554 |
| *Mechanical complication type*  VSR  LVFWR  PMR  VSR+LVFWR | 87 (49.7%)  49 (28.0%)  35 (20.0%)  4 (2.3%) | 52 (46.8%)  33 (29.7%)  25 (22.5%)  1 (0.9%) | 35 (54.7%)  16 (25.0%)  10 (15.6%)  3 (4.7%) | 0.330 |
| *Treatment type*  Surgical  Percutaneous  Hybrid/Staged  Conservative | 141 (80.6%)  6 (3.4%)  6 (3.4%)  22 (12.6%) | 90 (81.1%)  4 (3.6%)  5 (4.5%)  12 (10.8%) | 51 (79.7%)  2 (3.1%)  1 (1.6%)  10 (15.6%) | 0.613 |
| *Urgency^b^*  Elective  Urgent  Emergent  Salvage | 16 (10.5%)  68 (44.4%)  43 (28.1%)  26 (17.0%) | 7 (7.1%)  48 (48.5%)  25 (25.3%)  19 (19.2%) | 9 (16.7%)  20 (37.0%)  18 (33.3%)  7 (13.0%) | 0.126 |
| Symptoms to admission *(hours)* | 72.0 [12.0-120.0] | 72.0 [12.0-120.0] | 72.0 [12.0-118.1] | 0.840 |
| Symptoms to diagnosis *(hours)* | 72.0 [24.0-120.0] | 72.0 [24.0-120.0] | 72.0 [24.0-122.5] | 0.798 |
| Diagnosis to operation *(hours)^b^* | 60.0 [4.0-117.6] | 60.0 [5.0-120.0] | 42.0 [3.75-110.4] | 0.360 |

^a^Patients undergone coronarography (n=153). ^b^Operated patients (n=153). AMI: acute myocardial infarction; CABG: coronary artery bypass grafting; ECMO: extra-corporeal membrane oxygenation; LVFWR: left ventricular free-wall rupture; NSTEMI: non-ST-elevation MI; NYHA: New York Heart Association; PMR: papillary muscle rupture; STEMI: ST-elevation MI; VSR: ventricular septal rupture.

**Suppl. Table 2. Operative and perioperative data for operated patients (after imputation).**

| **Variables** | **Patients**  (n=153) | **Pre-COVID-19**  (n=99) | **COVID-19 era**  (n=54) | ***p*-value** |
| --- | --- | --- | --- | --- |
| CPB^a^ | 134 (91.2%) | 87 (91.6%) | 47 (90.4%) | 0.497 |
| CPB time^a^ *(min)* | 144.5 [102.0-170.3] | 136.5 [102.0-165.0] | 160.0 [126.0-174.0] | ***0.044*** |
| Aortic cross-clamp^a^ | 123 (83.7%) | 77 (81.1%) | 46 (88.5%) | 0.247 |
| Aortic cross-clamp time^a^ *(min)* | 100.0 [80.0-117.0] | 97.1 [74.3-115.5] | 102.0 [87.0-120.0] | 0.383 |
| Concomitant CABG^a^ | 64 (43.5%) | 38 (40.0%) | 26 (50.0%) | 0.242 |
| *Postoperative MCS*^b^  IABP  ECMO  Impella  Temporary RVAD | 86 (58.5%)  42 (28.6%)  8 (5.4%)  1 (0.7%) | 55 (57.9%)  27 (28.4%)  6 (6.3%)  1 (1.1%) | 31 (59.6%)  15 (28.8%)  2 (3.8%)  0 (0.0%) | 0.840  0.291  0.528  >0.999 |
| Postoperative inotropes^b^ | 123 (83.7%) | 76 (80.0%) | 47 (90.4%) | 0.103 |
| Re-thoracotomy for bleeding^b^ | 29 (19.7%) | 17 (17.9%) | 12 (23.1%) | 0.450 |
| Reoperation^b^ | 26 (17.7%) | 17 (17.9%) | 9 (17.3%) | 0.929 |
| CVVHDF^b^ | 45 (30.6%) | 27 (28.4%) | 18 (34.6%) | 0.436 |
| Ventilation time^c^ *(hours)* | 91.0 [23.5-148.1] | 86.0 [21.0-161.2] | 93.5 [28.0-148.0] | 0.575 |
| Intensive care unit stay^c^ *(hours)* | 144.0 [56.0-240.0] | 100.0 [57.0-178.9] | 182.9 [59.3-264.0] | 0.108 |
| Hospital stay^c^ *(days)* | 14.5 [10.0-27.0] | 14.0 [9.5-25.0] | 17.0 [12.5-31.0] | 0.130 |

^a^Patients undergone surgery (n=147). ^b^Operative survivors (n=147). ^c^Hospital survivors (n=64).

CABG: coronary artery bypass grafting; CPB: cardio-pulmonary bypass; CVVHDF: continuous veno-venous hemodiafiltration; ECMO: extra-corporeal membrane oxygenation; IABP: intra-aortic balloon pump; MCS: mechanical circulatory support; RVAD: right ventricular assist device.

**Suppl. Table 3. In-hospital mortality according to different subgroups**

| **Subgroups** | **Patients**  (n=175) | ***p*-value** | **Pre-COVID-19**  (n=111) | **COVID-19 era**  (n=64) | ***p*-value** |
| --- | --- | --- | --- | --- | --- |
| ***Mechanical complication type*** | | | | | |
| VSR (n=91)^a^ | 50 (54.9%) | ***0.003*** | 31 (58.5%) | 19 (50.0%) | 0.422 |
| LVFWR (n=53)^a^ | 32 (60.4%) |  | 21 (61.8%) | 11 (57.9%) | 0.782 |
| PMR (n=35) | 8 (22.9%) |  | 6 (24.0%) | 2 (20.0%) | >0.999 |
| ***Treatment type*** | | | | | |
| Surgical (n=141) | 62 (44.0%) | ***0.001*** | 44 (48.9%) | 18 (35.3%) | 0.118 |
| Percutaneous (n=6) | 4 (66.7%) |  | 2 (50.0%) | 2 (100.0%) | 0.467 |
| Hybrid/Staged (n=6) | 3 (50.0%) |  | 2 (40.0%) | 1 (100.0%) | >0.999 |
| Conservative (n=22) | 20 (90.9%) |  | 10 (83.3%) | 10 (100.0%) | 0.481 |
| ***Overall*** | | | | | |
| Intraoperative (n=153) | 6 (3.9%) | | 4 (4.0%) | 2 (3.7%) | >0.999 |
| All patients | 89 (50.9%) | | 58 (52.3%) | 31 (48.4%) | 0.627 |

^a^Four patients had concomitant VSR and LVFWR. LVFWR: left ventricular free-wall rupture; PMR: papillary muscle rupture; VSR: ventricular septal rupture.

**Suppl. Table 4. Number of cases diagnosed each year according to different countries.**

| **Country** | **Number of centers** | **Pre-COVID-19** | | **COVID-19 era** | **Change** |
| --- | --- | --- | --- | --- | --- |
|  |  | **2018-2019** | **2019-2020** | **2020-2021** |  |
| Austria | 1 | 2 | 2 | 4 | + 100.0% |
| France | 1 | 3 | 7 | 3 | - 40.0% |
| Germany | 4 | 16 | 22 | 14 | - 26.3% |
| Italy | 10 | 19 | 18 | 31 | + 66.8% |
| Poland | 1 | 9 | 8 | 8 | - 5.9% |
| The Netherlands | 1 | 3 | 2 | 4 | + 60.0% |
